# A Secure Pseudonym-Based Conditional Privacy-Preservation Authentication Scheme in Vehicular Ad Hoc Networks

**DOI:** 10.3390/s22051696

**Published:** 2022-02-22

**Authors:** Mahmood A. Al-Shareeda, Mohammed Anbar, Selvakumar Manickam, Iznan H. Hasbullah

**Affiliations:** National Advanced IPv6 Centre (NAv6), Universiti Sains Malaysia, USM, Gelugor 11800, Penang, Malaysia; m.alshareeda@nav6.usm.my (M.A.A.-S.); selva@usm.my (S.M.); iznan@nav6.usm.my (I.H.H.)

**Keywords:** Vehicular Ad hoc Networks (VANETs), security and privacy requirements, random oracle model, pseudonym identity scheme, Elliptic Curve Cryptography (ECC)

## Abstract

Existing identity-based schemes utilized in Vehicular Ad hoc Networks (VANETs) rely on roadside units to offer conditional privacy-preservation authentication and are vulnerable to insider attacks. Achieving rapid message signing and verification for authentication is challenging due to complex operations, such as bilinear pairs. This paper proposes a secure pseudonym-based conditional privacy-persevering authentication scheme for communication security in VANETs. The Elliptic Curve Cryptography (ECC) and secure hash cryptographic function were used in the proposed scheme for signing and verifying messages. After a vehicle receives a significant amount of pseudo-IDs and the corresponding signature key from the Trusted Authority (TA), it uses them to sign a message during the broadcasting process. Thus, the proposed scheme requires each vehicle to check all the broadcasting messages received. Besides, in the proposed scheme, the TA can revoke misbehaving vehicles from continuously broadcasting signed messages, thus preventing insider attacks. The security analysis proved that the proposed scheme fulfilled the security requirements, including identity privacy-preservation, message integrity and authenticity, unlinkability, and traceability. The proposed scheme also withstood common security attacks such as man-in-the-middle, impersonation, modification, and replay attacks. Besides, our scheme was resistant against an adaptive chosen-message attack under the random oracle model. Furthermore, our scheme did not employ bilinear pairing operations; therefore, the performance analysis and comparison showed a lower resulting overhead than other identity-based schemes. The computation costs of the message signing, individual signature authentication, and batch signature authentication were reduced by 49%, 33.3%, and 90.2%, respectively.

## 1. Introduction

In recent years, the Vehicular Ad hoc Network (VANET) has been attracting more and more attention from academia and industry [1,2]. According to a report published in 2015 [3,4], around 1800 fatalities and more than 20,000 injuries were due to road accidents annually in the United Kingdom. Therefore, the VANET, one of the cornerstone technologies of the Intelligent Transport System (ITS), is expected to help reduce traffic accidents [5,6].

**Figure 1:**
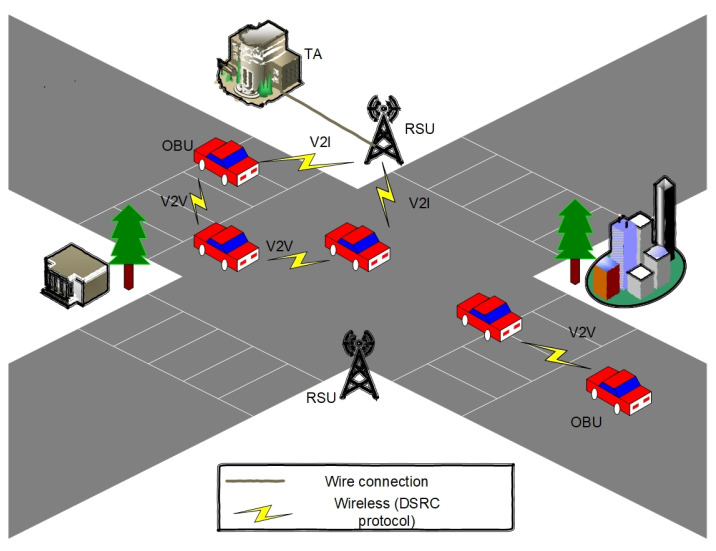
The structure of the VANET.

VANETs are an emerging type of Mobile Ad hoc Network (MANET), where the vehicle is considered a mobile node [7]. The VANET typically comprises three components; a Trusted Authority (TA), some fixed Roadside Unit (RSU), and many mobile Onboard Units (OBUs). As presented in Figure 1, a vehicle equipped with an OBU communicates with others via Vehicle-to-Vehicle (V2V) or with the RSU via Vehicle-to-Infrastructure (V2I) communications.

More specifically, driving safety and efficiency improvement are the main goals of ITS research, making VANETs a promising technology. Nevertheless, the advantages are out-weight by issues with security, privacy-preservation, and performance efficiency. Therefore, these challenges should be carefully considered in VANETs [8,9,10,11,12]. The security issue is crucial in V2V and V2I communications. The open nature of the transmission medium in VANETs is susceptible to security attacks [13,14,15], i.e., attackers can replay, modify, intercept, and impersonate transmitted messages in VANETs. Therefore, every receiver must check the authenticity and integrity of all received messages before accepting them. In addition, privacy preservation is also a fundamental requirement. In VANETs, attackers may discover the vehicle’s identity and trace its journey paths by dissecting captured messages. Therefore, anonymous communication is needed to preserve privacy and support drivers’ unlinkability requirements. Finally, performance efficiency is vital in V2V and V2I communications, apart from the security and privacy requirements.

Several scholars have proposed to address the security, privacy, and performance efficiency for the VANET system. However, some existing identity schemes have several limitations: (i) using time-consuming operations based on the bilinear pair; (ii) susceptible to an insider attack; (iii) only the vehicle’s message is verified by the RSU. As a result, this renders the whole system to be exposed and insecure.

Therefore, this paper aimed to cope with these three limitations arising from the existing identity schemes by generating lists of pseudonym-IDs and the corresponding signature keys by the TA. The main contribution of this paper is a secure pseudonym-based conditional privacy-preservation authentication scheme based on Elliptic Curve Cryptography (ECC).

The proposed scheme’s novelty is that: (i) it can sign and verify messages without relying on the online RSU for verification; (ii) the proposed scheme does not use the RSU during the mutual authentication process, thereby the TA issues and preloads the pool of pseudonym-IDs and the corresponding signature keys into the vehicle; (iii) the TA can revoke attackers’ certificates to prevent the continuous broadcast of fake signed messages.

The rest of the paper is structured as follows. The review of existing works is in Section 2. Section 3 presents the design of our scheme. Section 5 gives an illustrative example of the proposed scheme, followed by an in-depth discussion of the proposed scheme for VANETs in Section 4. Section 6 presents the security proof and analysis of the proposed scheme. In Section 7, we discuss the performance of the proposed scheme and a comparison with several existing schemes. Finally, Section 8 concludes this paper.

## 2. Related Work

In order to mitigate the burden of preloading several key pairs and their corresponding certificates from the common Public Key Infrastructure (PKI), in 1984, Shamir introduced the Identity (ID) approach [16]. This ID eliminated the need for key pairs and their corresponding certificates with the PKI due to not utilizing any certificate for verifying messages, thus decreasing the overhead generated from the messages containing certificates. Consequently, several studies have proposed ID-based schemes for communication security. In the following subsection, we classify the ID-based schemes in three ways.

### 2.1. ID Bilinear Pair Based

Zhang et al. [17,18] utilized the vehicle’s identity in which a vehicle is not required to preload a pool of key pairs and the corresponding certificates, eliminating the need for large storage, therefore reducing the overall processing overhead. Additionally, it mitigates the need to manage certificates and a CRL. Jiang et al. [19] suggested a Binary Authentication Tree (BAT) by using an ID-based scheme for V2I communication in VANETs. Huang et al. [20] suggested leveraging an ID-based scheme, called PACP, which relies on utilizing pseudonyms instead of the original identities, providing conditional privacy-preservation in VANETs. Chim et al. [21] and Lee and Lai [22] highlighted that the schemes proposed in [17,18] are not able to satisfy the traceability requirement. Besides, these schemes are vulnerable to impersonation and replay attacks. Lee and Lai [22] proposed an enhanced authentication scheme to secure communication and fulfill high-performance efficiency in VANETs. Horng et al. [23] pointed out that the scheme in [21] is vulnerable to security attacks such as impersonation and that an attacker can mimic an authorized vehicle for broadcasting bogus messages in VANETs. Therefore, Horng et al. [23] suggested a scheme named SPECS to enhance the scheme’s limitations [21]. Jianhong et al. [24] pointed out many security limitations in the scheme by Lee and Lai [22]. For instance, it cannot satisfy the requirements of non-repudiation and traceability and it cannot withstand attacks, such as replay attacks. In order to address the limitations in the scheme of Lee and Lai [22], Jianhong et al. [24] proposed an enhanced authentication scheme for communication security in VANETs.

ID-bilinear-pair-based schemes [17,18,19,20,21,22,23,24] utilize the bilinear pairing operations in their schemes. However, these schemes have a high overhead in terms of performance efficiency, owing to the time-consuming operation of the bilinear pair in VANETs.

### 2.2. ID Vulnerable to Insider Attack Based

He et al. [25] suggested an authentication scheme established on conditional privacy preservation for communication security in VANETs that does not utilize bilinear pairing operations during message signing and verification. For instance, in the scheme of He et al. [25], the system’s master key (TA) is preloaded and saved on the TPD of the vehicle and remains there for a long time. However, if an insider attacker compromises one vehicle, the entire VANET system will be vulnerable and insecure. The TA cannot revoke the compromised vehicle’s certificate to prevent it from being in the system. Therefore, the scheme by He et al. [25] does not satisfy the revocation requirement. Zhong et al. [26] structured a security and privacy scheme for secure service provision, accounting for messages’ security and users’ privacy in VANETs. Lo and Tasi [27] proposed an authentication scheme based on conditional privacy preservation for communication security in VANETs by adopting an ID-based scheme using ECC. Wu et al. [28] designed the concept of location to propose an authentication scheme based on conditional privacy preservation without using the operation of the bilinear pairing and TPD in VANETs. Xie et al. [29] proposed an authentication scheme based on conditional privacy preservation, which utilizes ID-based signatures to guarantee messages’ reliability and integrity in VANETs.

In ID vulnerable-to-insider-attack-based schemes [25,26,27,28,29], when a vehicle is transmitting false messages, the TA has the ability to trace this vehicle, but does not have the ability to revoke it for broadcasting these messages. Furthermore, an insider attacker has the ability to possibly disclose the vehicle’s identity, since the attacker has the key pairs of the TA. Thus, none of these schemes satisfy the revocation and privacy-preservation requirements in VANET.

### 2.3. ID RSU Authentication Based

Cui et al. [30] introduced a secure privacy-preservation authentication scheme based on ECC in VANETs. A cuckoo filter and binary search methods were used in this scheme to enhance the success rate of batch signature authentication. Zhong et al. [31] suggested an authentication scheme based on conditional privacy preservation, which utilizes the list of registration rather than the list of revocation to decrease the overhead of the system in terms of communication cost.

ID-RSU-authentication-based schemes [30,31] rely on RSUs to authenticate the traffic-related messages and then broadcast authentic and rogue vehicles lists with the notification issues. Therefore, the vehicle will wait for these issues before checking the validity of the signer, which increases the overhead.

In this paper, we propose a secure pseudonym-based conditional privacy-preservation authentication scheme to cope with the above-mentioned issues. It utilizes ECC rather than the bilinear pair operations to reduce the overhead of the system in terms of performance efficiency in ID-bilinear-pair-based schemes [17,18,19,20,21,22,23,24]. In addition, the authentic sender signs the message by utilizing a signature generated by the TA during the registration phase, and this process assists in coping with the flaws in ID vulnerable-to-insider-attack-based schemes [25,26,27,28,29]. Unlike the ID-RSU-authentication-based schemes [30,31], the proposed scheme relies on each vehicle checking the received messages.

## 3. Preliminaries

In this section, the network model, as well as the security requirements of the proposed scheme are presented. Besides, the mathematical tool used in this work is described as well.

### 3.1. Network Model

The network model of the proposed scheme consisted of three components, the TA, RSU, and OBU:

- TA: The TAs are trusted parties in VANETs with high resources such as computation and communication. The TA issues the system’s public parameters, pseudo-ID, and the private keys for each vehicle and transmits them to each respective vehicle;
- RSU: The RSU is a wireless base station deployed on the road as a brigade interface between the TA and OBUs. The RSU connects with the TA by wired technology and connects with vehicles by wireless technology;
- OBU: Each vehicle is fit with an On-Board Unit (OBU), enabling the vehicle to process, receive, and broadcast messages in the VANET. Each OBU is equipped with a Tamper-Proof Device (TPD) that is usually utilized to keep secrets. Therefore, it is difficult for any adversary to obtain the information stored in the TPD.

### 3.2. Security Requirements

The proposed scheme must fulfill all security and privacy requirements to achieve V2V and V2I communication security in VANETs. The security and privacy requirements are as follows:

- Authentication and integrity: The vehicle or RSU must be able to identify any alteration of the received message and must have the ability to authenticate the integrity and validity of the received messages to ensure communication security;
- Identity privacy preservation: An attacker must not have the ability to reveal the vehicle’s identity by capturing multiple messages transmitted by it. Therefore, the vehicle’s identity must remain anonymous to other legal and illegal nodes to ensure users’ privacy;
- Traceability: The TA must have the ability to reveal the vehicle’s identity from its message in case of any misbehavior to prevent misbehaving vehicles from denying their responsibility for disrupting the system by broadcasting false messages to other registered vehicles;
- Unlinkability: The misbehaving vehicles and RSUs cannot link two or more messages transmitted by the same source to ensure privacy preservation.

### 3.3. Adversary Model

A better understanding of adversity attacks against VANETs is needed. The following attack types should be resisted in the proposed scheme on VANETs:

- Replay attacks. Malicious nodes replay the previously generated legitimate signature to the recipient;
- Modification attacks. Malicious nodes alter authentic messages and broadcast them to other users [32];
- Impersonation attacks. Malicious nodes impersonate an authentic node and broadcast a legitimate message to other nodes;
- Man-in-the-middle attacks. Malicious nodes intercept two sides of the communication and perform data tampering and sniffing [33,34].

### 3.4. Elliptic Curve Cryptography

Elliptic Curve Cryptography (ECC) [35] is a tool used in the security algorithms’ design and digital signatures to secure communications. Due to the length of the smaller key and the same security level contrast with other encryption tools, ECC is commonly utilized in cryptography.

**Definition** **1.** *Elliptic curve: Consider that the large prime value p is the order of $F_{p}$ and $F_{p}$ is a finite field. The equation of an elliptic curve E is determined as $y^{2}$ = $x^{3}$ + ax + b mod p, where a, b∈$F_{p}$.*

*There is an additive group $G_{q}$ identified on E, the order of which is q, and the generator is P. Let O be an infinity point:*

– Scalar multiplication. Denote P ∈$G_{q}$, n ∈$Z_{q}^{*}$, then the scalar multiplication is L · P = P + P + P +…+P (for all the L times).

**Definition** **2.** *Computational Diffie–Hellman Problem (CDHP): There are two random points P, Q∈G, where P = yP, Q = xP, x, y are unknown integers, and it is impossible to calculate xyP.*

**Definition** **3.** *Elliptic Curve Discrete Logarithm (ECDL) problem: Given two random points P, Q∈G, and Q = x·P, it is impossible to calculate x from Q in the polynomial time t.*

## 4. The Proposed Scheme

**Figure 2:**
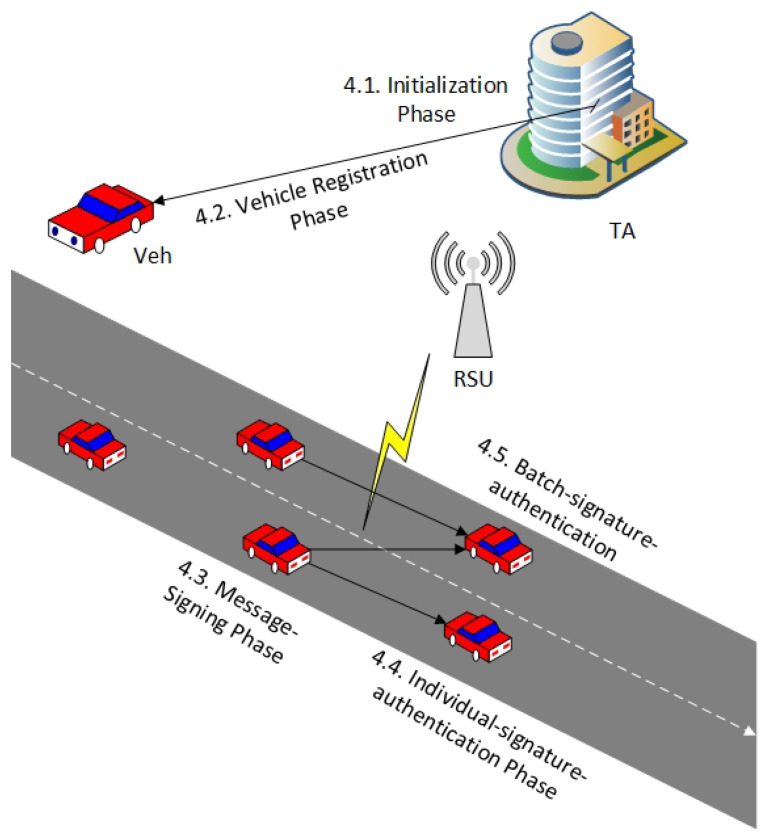
Overall flowchart of the proposed scheme.

Security and privacy are significant challenges that need to be carefully faced in VANET communication. This paper proposes a conditional privacy persevering based on mutual authentication scheme to fulfill the security and privacy requirements and reduce the system’s overhead. The secure pseudonym-based scheme means that the proposed scheme satisfies all security and piracy requirements mentioned (Section 3.2) and resists common security attacks, especially insider attacks. The proposed scheme consists of five phases: initialization, vehicle registration, message signing, individual signature authentication, and batch signature authentication, as shown in Figure 2.

The behavior of the overall system is as follows. The first phase is initialization, where the TA is responsible for generating and preloading the public parameters of the system based on an elliptic curve. The second phase is vehicle registration, where the TA is responsible for generating and preloading the list of pseudonym-IDs and signature keys to each participating registered vehicle in the VANET. The third phase is message signing, where the registered vehicle signs each traffic message by using randomly the pseudonym-ID and the signature key before broadcasting. The fourth phase is individual signature authentication, where the receiving vehicle should verify the validity and authenticity of the message before accepting. The fifth phase is batch signature authentication, where the verified vehicle has the ability to check a large number of messages simultaneously. Furthermore, when receiving a report about a malicious vehicle, the TA is responsible for tracing and revoking it. After all pseudonym-IDs have expired, the TA does not update the new pseudonym-ID list to avoid it being utilized for additional applications and services in the VANET. Table 1 presents the notation utilized and their definitions in the following phases.

### 4.1. Initialization

The TA executes the initialization parameter of the public system in the following steps:

- The TA sets the chosen elliptic curve *E* determined by the non-singular equation ($y^{2}=x^{3}+ax+b$ mod *p*), where $a,b\in F_{p}$ and *p* is a large prime number;
- The TA chooses a point *P* on $E_{p}\left(a,b\right)$ as an adaptive group generator *G* of prime order *q*;
- The TA selects the private key *s*∈$Z_{q}^{*}$ of the system and computes the respective public key $P_{pub}$ = sP of the system;
- The TA selects three secure cryptographic hash functions $h_{1}:G\to Z_{q}^{*}$$h_{2}:{\left\{0,1\right\}}^{*}\times {\left\{0,1\right\}}^{*}\times G\to Z_{q}^{*}$$h_{3}:{\left\{0,1\right\}}^{*}\to Z_{q}^{*}$;
- The TA publishes the functions and the public parameters of the system to all RSUs via public channels.

### 4.2. Vehicle Registration

The TA registers the vehicle as follows:

- The owner of the vehicle submits personal information including the identity $ID_{vi}$ and password PW to the TA through a secure communication channel;
- After the personal information is received, the TA first starts the authenticity of $ID_{vi}$;
- After checking the validity of $ID_{vi}$, the TA chooses n random secret values $\zeta_{l}$∈$Z_{q}^{*}$, where l=1:n, and calculates a family of unlinkable pseudo-IDs $LPID_{i}=<pid_{il},\ldots ,pid_{in}>$ as follows:
  (1) $$\begin{array}{cc} pid_{in} & =<pid_{il}^{1},pid_{il}^{2}>\\ \; & =<\zeta_{l}P,ID_{vi}⊕h_{1}\left(\zeta_{l}P_{pub}\right)> \end{array}$$
  where *l* = 1,2, …*n*;
- For each pseudo-ID $pid_{il}$∈$LPID_{i}$, l=1:n, the TA calculates the respective signature key SK as follows and organizes $LSK_{i}=<sk_{il},\ldots ,sk_{in}>$:
  (2) $$\begin{array}{c} sk_{il}=s\cdot h_{2}(pid_{il}^{1}||pid_{il}^{2}); \end{array}$$
- The TA then transmits the n of $\zeta_{l}$, $LPID_{i}$, and $LSK_{i}$ to the vehicle via a secret technology.

The process of preloading as introduced in [26,36] is to guarantee the requirements of the security and privacy of $\zeta_{l}$, the pseudo-ID, and the signature keys for the proposed scheme. The TA preloads a new list of $\zeta_{l}$, the pseudo-ID, and the signature keys that are utilized for a short time for each vehicle moving in a VANET close to the expiration time; they are renewed with a new pseudo-ID and signature key pool.

Our previous study [37] was based on the RSU executing the authentication process by issuing and preloading a pool of pseudonym-IDs and the corresponding signature keys into each registered vehicle. However, the disadvantages of RSU utilization are: (i) once a single RSU is compromised, as a result, the whole system becomes insecure; (ii) RSUs are expensive in terms of installation and maintenance; (iii) adding a TPD to both the OBU and the RSU makes the system even more costly. Besides, our previous study [37] depended on generating several keys to each domain, which makes the key exchange complete.

Therefore, this paper aimed to address these issues by issuing and preloading a pool of pseudonym-IDs and the corresponding signature keys from the TA. This was because the resource of the TA is high in terms of computation and communication costs. Hence, the proposed scheme does not use RSUs during the mutual authentication process. Besides, only the private key and public key of the TA are used to sign and verify the messages.

### 4.3. Message Signing

The signer (OBU or RSU) signs and broadcasts traffic-related messages $m_{i}$ to other vehicles in the VANET. A vehicle with pseudo-ID $pid_{in}$ receives a message $m_{i}$ and signs it by utilizing its signature keys $sk_{il}$ and the public parameter of the system. This is executed in the phases below:

- $OBU_{i}$ randomly chooses a pseudo-ID $pid_{in}$ with the respective $\zeta_{l}$ and $sk_{il}$;
- $OBU_{i}$ computes the message signature $\delta_{m_{i}}=sk_{il}+\zeta_{l}\cdot h_{3}(m_{i}\left||T\right)$, where *T* is the current timestamp;
- $OBU_{i}$ computes $Y_{i}=h_{3}(m_{i}||T)pid_{il}^{1}$;
- $OBU_{i}$ sets the authentic signature as $\sigma_{i}=\left\{\delta_{m_{i}},Y_{i}\right\}$ for $m_{i}$;
- Finally, the message signature tuple {$pid_{in}$, $m_{i}$, *T*, $\sigma_{i}$} is sent to the neighboring recipient.

### 4.4. Individual Signature Authentication

The main aim of this method was to verify only one message signature $\delta_{m_{i}}$ on traffic-related message $m_{i}$ by the recipient (OBU or RSU). Before accepting the message $m_{i}$, once having received a signed message $m_{i}$, the recipient would check the node authenticity and validity of the message. This guarantees that no illegitimate recipient is impersonating a legitimate recipient or sending fake messages. The recipient receives an authentic signature $\sigma_{i}=\left\{\delta_{m_{i}},Y_{i}\right\}$ on the traffic-related message $m_{i}$ from the vehicle with a pseudo-ID $pid_{in}$ in timestamp *T*, where i=1, and checks its authenticity and validity as below:

- Once the message signature tuple {$pid_{in}$, $m_{i}$, *T*, $\sigma_{i}$} is received, the OBU first verifies the validity of timestamp *T*. If (*T*>$T_{r}$ − $T_{▽}$), where $T_{r}$ is the time of receiving and $T_{▽}$ is the time of the predefined delay, then *T* is fresh. Otherwise, the message is rejected;
- The OBU utilizes the public parameter and functions of the system and authentic signature $\sigma_{i}=\left\{\delta_{m_{i}},Y_{i}\right\}$ on the message $m_{i}$. Therefore, if Equation (3) holds, the OBU accepts it.

(3) $$\delta_{m_{i}}P=h_{2}(pid_{il}^{1}||pid_{il}^{2})P_{pub}+Y_{i}$$

The proof of the correctness is as follows:

$$\begin{array}{cc} \; & L.H.S\left(\delta m_{i}\cdot P\right)\\ \; & =\left(sk_{il}+\zeta_{l}\cdot h_{3}\left(m||T\right)\right).P\\ \; & =\left(s\cdot h_{2}(pid_{il}^{1}||pid_{il}^{2})+\zeta_{l}\cdot h_{3}\left(m||T\right)\right)\cdot P\\ \; & =h_{2}(pid_{il}^{1}||pid_{il}^{2})s\cdot P+h_{3}\left(m||T\right)\zeta_{l}\cdot P\\ \; & =h_{2}(pid_{il}^{1}||pid_{il}^{2})P_{pub}+h_{3}\left(m||T\right)pid_{il}^{1}\\ \; & =h_{2}(pid_{il}^{1}||pid_{il}^{2})P_{pub}+Y_{i}\\ \; & =R.H.S \end{array}$$

Thus, the individual signature authentication correctness is accurate.

### 4.5. Batch Signature Authentication

The main aim of this method is to authenticate a multiple of messages signature $\delta_{m_{i}}=\left\{\delta_{m_{1}},\delta_{m_{2}},\delta_{m_{3}},\ldots ,\delta_{m_{n}}\right\}$ on n traffic-related messages $m_{i}=\left\{m_{1},m_{2},m_{3}\ldots ,m_{n}\right\}$ from n vehicles with n pseudo-ID $pid_{in}=\{pid_{i1},pid_{i2}$, $pid_{i3},\ldots ,$$pid_{in}\}$. The verifying recipient checks its authenticity and validity as shown in the following steps:

- The OBU checks the validity of timestamp *T*. If (*T*>$T_{r}$−$T_{▽}$), *T* is fresh. Otherwise, the message is rejected;
- The OBU utilizes the small exponent technique [23,38] to achieve security in the proposed scheme. The OBU issues a random value $\gamma_{i}$= {$\gamma_{1},\gamma_{2},\gamma_{3},\ldots ,\gamma_{n}$}, where $\gamma_{i}\in$$\left[1:2^{t}\right]$ and *t* is a small value;
- The OBU utilizes the following Equation (4) to accept them.

(4) $$\left(\sum_{i=1}^{n}\left(\gamma_{i}\cdot \delta_{m_{i}}\right)\right)\cdot P=\left(\sum_{i=1}^{n}(\gamma_{i}.h_{2}(pid_{il}^{1}||pid_{il}^{2})P_{pub})\right)+\sum_{i=1}^{n}\left(\gamma_{i}Y_{i}\right)$$

## 5. Illustrative Example

In this section, we describe an illustrative example of the five phases of the proposed scheme: initialization, vehicle registration, message signing, individual signature authentication, and batch signature authentication according to our simulation experiment (Section 7.1). The illustrative example of the proposed scheme is as follows.

### 5.1. Example of Initialization Phase

**Figure 3:**
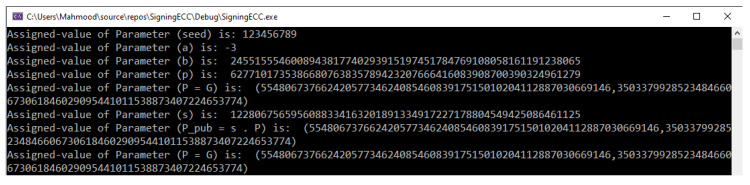
Input parameters and assigned values for the illustrative examples.

The first phase includes the initialization of the system’s public parameters and the generation of the secure key pairs by the TA component in the VANET system. Figure 3 shows the parameters and their assigned values used in the illustrative examples. These parameters were generated based on the NIST P-192 Curve.

### 5.2. Example of the Vehicle Registration Phase

**Figure 4:**
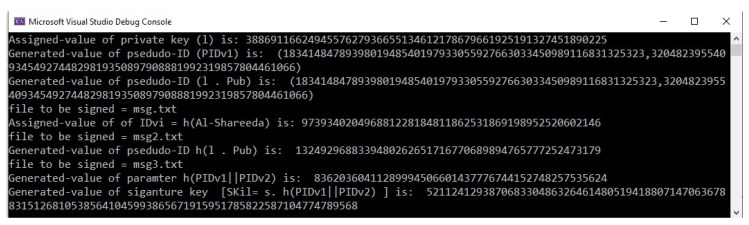
List of pseudonym-IDs and the corresponding signature keys.

The second phase includes the vehicle registration by the TA before the vehicle leaves the factory. The TA is responsible for issuing and preloading the list of pseudonym-IDs and the corresponding signature keys to each participating vehicle. Figure 4 shows one example of a list of pseudonym-IDs and the corresponding signature keys.

As mentioned in Equation (1), $pid_{il}^{2}=ID_{vi}⊕h_{1}\left(\zeta_{l}P_{pub}\right)$, where $ID_{vi}$ = 973934020496881228184811862531869198952520602146 and $h_{1}\left(\zeta_{l}P_{pub}\right)$ = 1324929688339480262651716770689894765777252473179. Therefore, the result of $pid_{il}^{2}$ is:
973934020496881228184811862531869198952520602146 ⊕1324929688339480262651716770689894765777252473179 =379900236377609805635535841290573035328518392697
where $pid_{il}^{2}$ is the pseudonym-ID of the vehicle, $ID_{vi}$ is the real identity of the vehicle, $\zeta_{l}$ is a random private key of $pid_{il}^{2}$, and $P_{pub}$ is the published key of the system (TA). All these parameters are based on an elliptic curve.

### 5.3. Example of the Message Signing Phase

**Figure 5:**
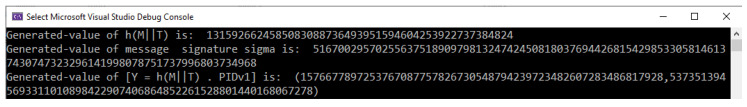
Broadcasting message signature tuple in the VANET.

After the vehicle has saved the list of signature keys and the corresponding signature keys, it is considered as an authenticated node and allowed to broadcast messages. Figure 5 shows the broadcasting message signature tuple in the VANET.

### 5.4. Example of the Individual Signature Authentication Phase

Upon receiving the message signature tuple, the verifier uses a scalar multiplication operation to check the freshness of the timestamp and the validity of the message. The verifier executes the following process:

$\sigma_{i}\cdot P=$ (2270327100600948043112723198985285564808416667064180454920,

1952967422112747467668372522590950986900866071665660895860)

### 5.5. Example of the Batch Signature Authentication Phase

Upon receiving several message signature tuples, the verifier checks all signatures simultaneously as follows:

$\sum_{i=1}^{n}\left(\sigma_{i}\cdot \gamma_{i}\right)\cdot P$ = (3108195267006925604593061313615253284225390209158609199161,

2944227777884750291423675754070844581166705394496268347467), where $\sigma_{i}=11$.

## 6. Security Proof and Analysis

This section evaluates the proposed scheme’s security proof, analysis, and comparison as follows.

### 6.1. Security Proof

Several scholars [25,30,31] have proposed the most secure signature algorithms that satisfy the random oracle model based on their scheme. This work was also needed to satisfy the random oracle model based on the renew procedure, pseudo-ID, and signature keys for the proposed scheme. Based on the network model and the ability of the malicious node, we show the security proof in the proposed scheme by identifying a game between attacker *A* and challenger *C*. When the game is won by attacker *A*, a legally forged signature can easily be returned. Consequently, if attacker *A* has negligible effectiveness, the proposed scheme is secure in the VANET.

**Theorem** **1.** *Under the random oracle model, the proposed scheme can be unforgeable against an adaptively selected message attack.*

**Proof.**  Suppose an attacker *A* can forge a legitimate message signature tuple {$pid_{in}$, $m_{i}$, *T*, $\sigma_{i}$} for the VANET; therefore, a challenger *C* could be issued to return the ECDL problem by working *A* as a subroutine with non-negligible probability.

Setup initialization phase: Challenger *C* first sets value *s*∈$Z_{q}^{*}$ chosen randomly as the system’s master key and calculates $P_{pub}$ = sP as the system’s public key. Hence, *C* broadcasts the system’s functions and public parameters to *A*.

$h_{1}$-oracle. *C* initializes $h_{list_{1}}$ in the form of $\left(\alpha ,\tau h_{1}\right)$. Once *A* receives a message in the form of (α), *C* tests whether (α) is in $h_{list_{1}}$, and if it exists, *C* sends $\left(\tau h_{1}=h\left(\alpha \right)\right)$ to *A*. Otherwise, *C* sets the chosen value $\tau h_{1}\in Z_{q}^{*}$ randomly and adds $\left(\alpha ,\tau h_{1}\right)$ into $h_{list_{1}}$. Then, *A* broadcasts $\tau h_{1}=h\left(\alpha \right)$ to *C*.

$h_{2}$-oracle. *C* initializes $h_{list_{2}}$ in the form of $\left(pid_{il}^{1},pid_{il}^{2},\tau h_{2}\right)$. After *A* receives the message in the form of $\left(pid_{il}^{1},pid_{il}^{2}\right)$, *C* tests whether $\left(pid_{il}^{1},pid_{il}^{2}\right)$ is in $h_{list_{2}}$, and if it exists, *C* broadcasts $(\tau h_{2}=h(pid_{il}^{1}||pid_{il}^{2}||\tau h_{2})$ to *A*. Otherwise, *C* sets the chosen value $\tau h_{2}\in Z_{q}^{*}$ randomly and adds $\left(pid_{il}^{1},pid_{il}^{2},\tau h_{2}\right)$ into $h_{list_{2}}$. Then, *A* broadcasts $\tau h_{2}=h(pid_{il}^{1}||pid_{il}^{2}||\tau h_{2})$ to *C*.

$h_{3}$-oracle. *C* initializes $h_{list_{3}}$ in the form of $\left(m_{i},T,\tau h_{3}\right)$. After *A* receives the message in the form of $(m_{i},T$), *C* tests whether $\left(m_{i},T\right)$ is in $h_{list_{3}}$, and if it exists, *C* sends $(\tau h_{3}=h(m_{i}||T||\tau h_{3})$ to *A*. Otherwise, *C* chooses $\tau h_{3}\in Z_{q}^{*}$ randomly and puts $\left(m_{i},T,\tau h_{3}\right)$ into $h_{list_{3}}$. Then, *A* sends $\tau h_{3}=h(m_{i}||T||\tau h_{3})$ to *C*. □

Sign oracle:

Upon receiving a sign request from *A*, *C* calculates three random numbers, $h_{i,2}$; $h_{i,3}$; $\delta_{m,i}\in Z_{q}^{*}$ and a random point $pid_{il}^{2}$∈*G*. Then, *C* computes $pid_{il}^{1}$∈ = ($\delta_{m,i}P-h_{i,2}P_{pub}/h_{i,3}$). *C* puts $\left(pid_{il}^{1},pid_{il}^{2},\tau h_{2}\right)$ into $h_{list_{2}}$ and $\left(m_{i},T\right)$ into $h_{list_{3}}$. Finally, *C* generates a message signature tuple {$pid_{in}$, $m_{i}$, *T*, $\sigma_{i}$} and transmits it to *A*, where $pid_{in}=pid_{il}^{1},pid_{il}^{2}$. The reply is a legal sign oracle due to the message signature tuple {$pid_{in}$, $m_{i}$, *T*, $\sigma_{i}$} achieving the following equation:

$$\begin{array}{cc} \delta m_{i}\cdot P & =h_{i,2}P_{pub}+Y_{i}\\ & whereY_{i}=h_{i,3}pid_{il}^{1}\\ \delta m_{i}\cdot P & =h_{i,2}P_{pub}+h_{i,3}pid_{il}^{1}\\ \; & =h_{i,2}P_{pub}+\left(\delta m_{i}P-h_{i,2}P_{pub}\right)=\delta m_{i}\cdot P \end{array}$$

Output: Lastly, *A* results in a message signature tuple {$pid_{in}$, $m_{i}$, *T*, $\sigma_{i}$}. *C* tests this message using the following equation:

(5) $$\delta_{m_{i}}P=h_{i,2}P_{pub}+Y_{i}$$

If Equation (5) does not hold, *C* ends the game.

According to the cross lemma, *A* can output another message signature tuple {$pid_{in}$, $m_{i}$, *T*$\sigma_{i}^{*}$} that achieves the following equation:

(6) $$\delta_{m_{i}}^{*}P=h_{i,2}^{*}P_{pub}+Y_{i}$$

According to Equations (5) and (6), we can obtain:

$$\begin{array}{cc} (\delta_{m_{i}}-\delta_{m_{i}}^{*})P & =\delta_{m_{i}}P-\delta_{m_{i}}^{*}P\\ \; & =\left(h_{i,2}P_{pub}+Y_{i}\right)-\left(h_{i,2}^{*}P_{pub}+Y_{i}\right)\\ \; & =h_{i,2}P_{pub}-h_{i,2}^{*}P_{pub}\\ \; & =\left(h_{i,2}-h_{i,2}^{*}\right)P_{pub}\\ \; & =(h_{i,2}-h_{i,2}^{*})sP \end{array}$$

Then, we can obtain $\left(\delta_{m_{i}}-\delta_{m_{i}}^{*}\right)=\left(h_{i,2}-h_{i,2}^{*}\right)$ s mod P.

*C* solves the ECDL problem by computing $\left(\delta_{m_{i}}-\delta_{m_{i}}^{*}\right).{\left(h_{i,2}-h_{i,2}^{*}\right)}^{-1}$. Nevertheless, under the random oracle model, owing to the ECDL problem difficulty with the non-negligible probability, the proposed scheme is resistant against an adaptively selected message attack.

### 6.2. Security Analysis

This subsection discusses the analyses of the proposed scheme that should achieve the security requirements according to Section 3.2 as follows.

**Figure 6:**
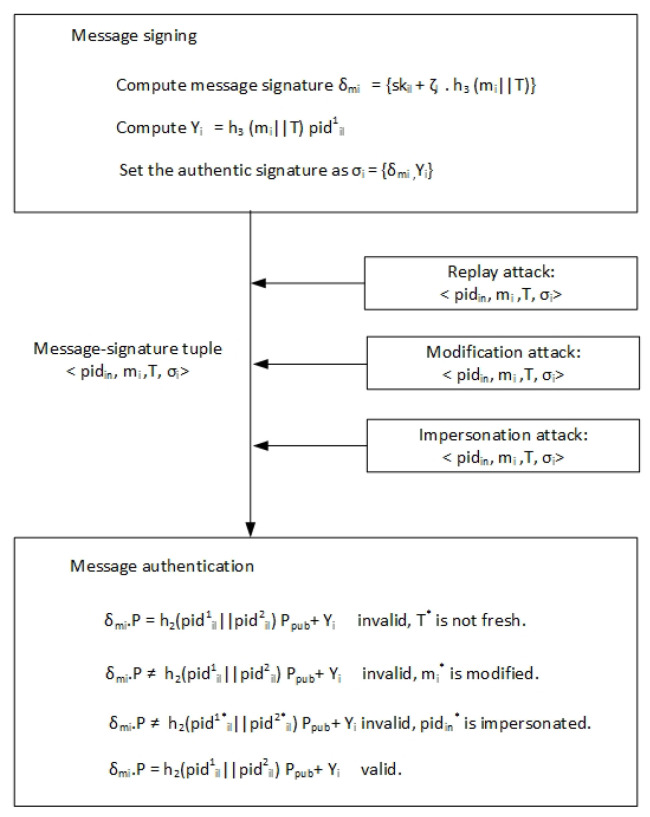
The process of the system resisting attacks.

- Authentication and integrity: Consistent with Theorem 1, no malicious node can return the ECDL problem and generate the legitimate signature; it is considered to be forged otherwise. In our scheme, the verifying recipient can test the authenticity and integrity of the message signature tuple {$pid_{in}$, $m_{i}$, *T*, $\sigma_{i}$} sent from the vehicle by checking the equation $\delta_{m_{i}}P=h_{2}(pid_{il}^{1}||pid_{il}^{2})P_{pub}+Y_{i}$ before accepting it. If verified and validated, the recipient accepts traffic-related message $m_{i}$; otherwise, the message is rejected. Thus, our scheme can satisfy messages’ authentication and integrity requirements;
- Identity privacy preservation: After the identity $ID_{vi}$ of a vehicle is received, the TA converts it to pseudo-ID $pid_{in}$ in the proposed scheme. The main purpose of this requirement is to support anonymous communication and preserve the driver’s privacy. The pseudo-ID $pid_{in}$ involves two secret values $\zeta_{l}$ and *s* selected randomly by the OBU and TA, respectively. It is impossible for an attacker to disclose identity $ID_{vi}$ from pseudo-ID $pid_{in}$ = $pid_{in}=<pid_{il}^{1},pid_{il}^{2}>$$=<\zeta_{l}P,ID_{vi}⊕h_{1}\left(\zeta_{l}P_{pub}\right)>$ of any vehicle without knowing $\zeta_{l}$ and *s*. Therefore, it cannot calculate $spid_{il}^{1}=s\zeta_{l}P$ from $P_{pub}=sP$ and $pid_{il}^{2}=\zeta_{l}P$ to obtain the identity $ID_{vi}$ of the vehicle because it is a difficult CDHP problem. Thus, the proposed scheme can satisfy the identity privacy-preservation requirement in the VANET;
- Traceability: If a malicious node broadcasts a bogus message, i.e., $m_{i}$ to participating vehicles to disrupt the system managing the road, the TA can revoke the malicious node’s identity after tracing him/her during traveling. Suppose a vehicle $V_{i}$ issues a false message $m_{i}$ and sends it to a vehicle $V_{j}$. The TA receives a report on the forged message $m_{i}$ from vehicle $V_{j}$. The TA verifies the pseudo-ID $pid_{in}$ on message $m_{i}$ for vehicle $V_{i}$ in its database registration list. When the pseudo-ID $pid_{in}$ is match stored, the TA uses its private key *s* to disclose the identity $ID_{vi}$ of vehicle $V_{i}$ by calculating the following:
  (7) $$\begin{array}{cc} ID_{vi} & =pid_{il}^{2}⊕h_{1}\left(s.pid_{il}^{1}\right)\\ \; & =h_{1}\left(\zeta_{l}.P_{pub}\right)⊕h_{1}\left(s.pid_{il}^{1}\right)\\ \; & =ID_{vi} \end{array}$$
  After tracing the vehicle’s identity, the TA revokes its database registration list, saves it in the Certificate Renovation List (CRL). The vehicle cannot send traffic-related messages in the VANET. Therefore, our scheme can satisfy the traceability requirement in the VANET;
- Unlinkability: Each message signature tuple {$pid_{in}$, $m_{i}$, *T*, $\sigma_{i}$} involves a pseudo-ID $pid_{in}=<pid_{il}^{1},pid_{il}^{2}>$, where $pid_{il}^{1}=\zeta_{l}\cdot P$ and $\zeta_{l}$∈$Z_{q}^{*}$ is a random secret value; therefore, the particular vehicle generates the different pseudo-ID in our scheme. Furthermore, since vehicles utilize different pseudo-IDs to sign every message $m_{i}$, an attacker cannot link multiple messages transmitted by the same source. Thus, the proposed scheme can satisfy the unlinkability requirement in the VANET;
- Security attack resistance: The proposed scheme can resist the common attacks as follows. Figure 6 shows the process of the system resisting replay, modification, and impersonation attacks;
  - Replay attacks. In the proposed scheme, timestamp *T* in the message signature tuple {$pid_{in}$, $m_{i}$, *T*, $\sigma_{i}$} allows the recipient to check the authenticity of the message $m_{i}$. Once the vehicle receives the message $m_{i}$, it verifies the freshness of the timestamp by verifying whether the inequalities (*T*>$T_{r}$−$T_{▽}$) hold. If it is fresh, the message $m_{i}$ is accepted; otherwise, the vehicle does not accept message $m_{i}$. The proposed scheme can detect the message $m_{i}$ replay in the VANET. Therefore, our scheme can withstand replay attacks in the VANET;
  - Modification attacks. An attacker cannot modify a message signature tuple {$pid_{in}$, $m_{i}$, *T*, $\sigma_{i}$} consistent with Theorem 1 since the vehicle can expose any alteration in the tuple by verifying the equation $\delta_{m_{i}}P=h_{2}(pid_{il}^{1}||pid_{il}^{2})P_{pub}+Y_{i}$. Therefore, the alteration probability of the signature for the message $m_{i}$ is minimal. Therefore, the proposed scheme can withstand modification attacks in the VANET;
  - Impersonation attacks. It is impossible for an attacker to forge a legitimate message signature tuple {$pid_{in}$, $m_{i}$, *T*, $\sigma_{i}$} consistent with Theorem 1 because the recipient verifies the authenticity of the tuple {$pid_{in}$, $m_{i}$, *T*, $\sigma_{i}$} by checking the equation $\delta_{m_{i}}P=h_{2}(pid_{il}^{1}||pid_{il}^{2})P_{pub}+Y_{i}$. The forged signature probability for message $m_{i}$ is trivial. Therefore, the proposed scheme can resist impersonation attacks in the VANET;
  - Man-In-The-Middle (MITM) attacks. In the proposed scheme, mutual authentication is executed among the nodes in the VANET. If an attacker tries an MITM attack, forged messages must link with the signer and the receiver. Nevertheless, consistent with Theorem 1, it is impossible for an attacker to launch this attack type. Therefore, the proposed scheme can resist MITM attacks in the VANET.

### 6.3. Security Comparison

We compared the performance of our scheme with other ID-based schemes. Table 2 presents the comparison results, where SC-1, SC-2, and SC-3 denote bilinear pair used, vulnerable to insider attacks, and RSU authentication, respectively.

As presented in Table 2, we know that none of them completely address all security issues such as bilinear pair used, vulnerability to an insider attack, and RSU authentication in their scheme. However, the proposed scheme addresses all security issues regarding identity-based schemes in VANETs.

## 7. Performance Analysis and Comparison

This section presents the experiment and the comparative performance analysis of the proposed scheme and other schemes in terms of computation and communication costs.

### 7.1. Experimental

**Figure 7:**
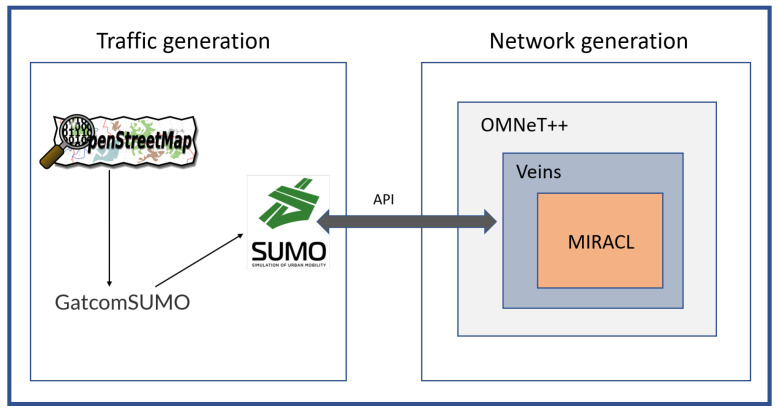
VANET simulation.

The simulation experiment of the proposed scheme includes two parts, namely network generation and road traffic generation. As shown in Figure 7, this paper used OMNeT++ [39], VEINS [40], MIRACL [41,42], OpenStreetMap [43], GatcomSUMO [44], and SUMO [45] to carry out the simulation experiments for VANETs. OMNeT++ is a modular, component-based C++ simulation library for communication networks. VEINS combines road traffic generation and network generation. MIRACL is a cryptographic library used to execute cryptography operations for algorithms. OpenStreetMap is the most prominent crowd-sourced web-based mapping platform. GatcomSUMO is a graphical application that simplifies VANET simulation, specifically the SUMO traffic and the OMNeT++ network generation. SUMO is a highly portable, multi-model traffic simulation. Table 3 presents the simulation experiment parameters.

For road traffic generation, each vehicle has some functional characteristics such as the minimum and maximum speed, dimension, and direction. These characteristics influence and restrict the mobility model.

**Figure 8:**
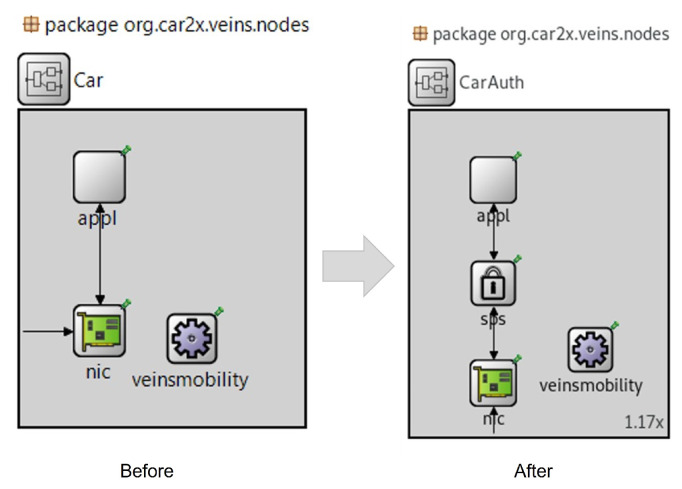
Signing and verifying messages in OMNeT++.

**Figure 9:**
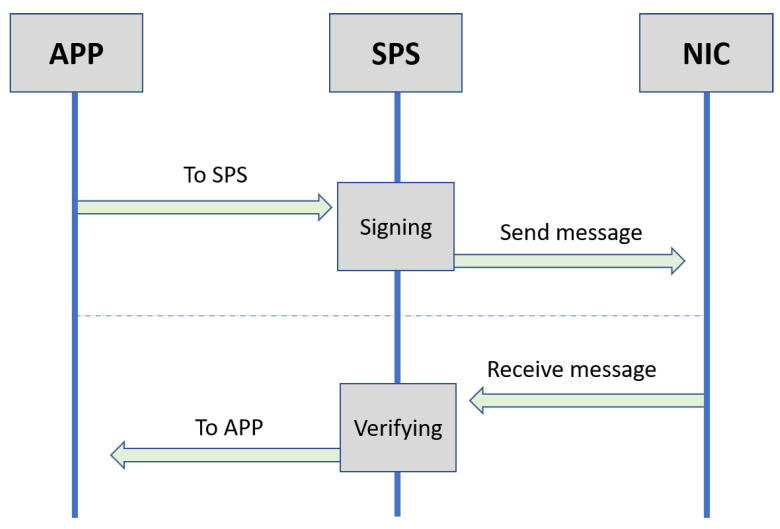
Data flow for sending and receiving messages.

In this work, the trip trajectory and mobility model were random, and the number of vehicles was constant. In the simulation experiment of the proposed scheme, the Security Processing Service (SPS) layer was added in each RSU and OBU on network simulators (VEINS/OMNeT++). The main reason behind the SPS layer used was to execute the process of signing and verifying messages that was higher than the MAC and physical layer and lower than the application layer, as shown in Figure 8. In the VANET communications, the data flow for sending and receiving messages during three layers, namely, the App, SPS, and NIC layers, is shown in Figure 9.

### 7.2. Computation Cost Analysis and Comparison

This subsection evaluates and compares the computation costs between the proposed scheme and the existing schemes. The notations for the executing time and time cost are as follows:

- $T_{bp}$ indicates the time needed to perform a bilinear pairing operation. Hence, the time cost of $T_{bp}$ is 5.811 ms;
- $T_{b.a}$ indicates the time needed to perform the point addition operation for the bilinear pairing in $G_{1}$. Hence, the time cost of $T_{b-a}$ is 0.0106 ms;
- $T_{b.sm}$ indicates the time needed to perform a small scalar multiplication operation for the bilinear pairing in $G_{1}$. Hence, the time cost of $T_{b.sm}$ is 0.1829 ms;
- $T_{b.m}$ indicates the time needed to perform a scalar multiplication operation for the bilinear pairing in $G_{1}$. Hence, the time cost of $T_{b.m}$ is 1.5654 ms;
- $T_{mtp}$ indicates the time needed to perform a map-to-point function for the bilinear pairing in $G_{1}$. Hence, the time cost of $T_{mtp}$ is 4.1724 ms;
- $T_{e-m}$ indicates the time needed to perform a scalar multiplication operation for ECC in an additive group *G*. Hence, the time cost of $T_{e-m}$ is 0.6718 ms;
- $T_{e-a}$ indicates the time needed to perform a point addition operation for ECC in an additive group *G*. Hence, the time cost of $T_{e-a}$ is 0.0031 ms;
- $T_{e.sm}$ indicates the time needed to perform a small scalar multiplication for ECC in *G*. Hence, the time cost of $T_{e.sm}$ is 0.0665 ms;
- $T_{h}$ indicates the time required to perform a secure hash cryptography function. Hence, the time cost of $T_{h}$ is 0.001 ms.

For easy measurement, let MSG, ISA, and BSA be the message signing generation, individual signature authentication, and batch signature authentication, respectively.

In He et al.’s scheme [25], three secure hash cryptography functions and three ECC-based scalar multiplication operations are needed during the MSG, resulting in a total cost of $3T_{e-m}+3T_{h}\approx 2.0184$ ms. This scheme involved two point-addition operations, two secure hash cryptography functions, and three scalar multiplication operations for ISA, resulting in a total cost of $3T_{e-m}+2T_{e-a}+2T_{h}\approx 2.0236$ ms. During the BSA, (2n) functions regarding secure hash cryptography, (2n−1) operations regarding point addition, (2n) operations regarding small scalar multiplication, and (n+2) operations regarding scalar multiplication are needed in this scheme; thus, the whole cost is $\left(n+2\right)T_{e-m}+\left(2n\right)T_{e-sm}+\left(2n-1\right)T_{e-a}+\left(2n\right)T_{h}\approx 0.6718n+1.3405$ ms. MSG includes a scalar multiplication and two secure hash cryptography functions in the proposed scheme, resulting in the whole cost being $1T_{e-m}+2T_{h}\approx 0.6738$ ms. Meanwhile, ISA includes two scalar multiplication, one secure hash cryptography, and one point addition operation in the proposed scheme, resulting in the whole cost being $2T_{e-m}+1T_{h}+2T_{e-a}\approx 1.3477$ ms. Finally, BSA includes two operations regarding scalar multiplication, (2n) operations regarding small scalar multiplication, (n+1) operations regarding point addition, and (*n*) functions regarding secure hash cryptography in the proposed scheme; thus, the whole cost is $2T_{e-m}+\left(2n\right)T_{e-sm}+\left(n+1\right)T_{e-a}+\left(n\right)T_{h}\approx 0.0737n+1.3467$ ms. We also measured the computation cost of other schemes’ MSG, ISA, and BSA using the same procedure, as tabulated in Table 4.

**Figure 10:**
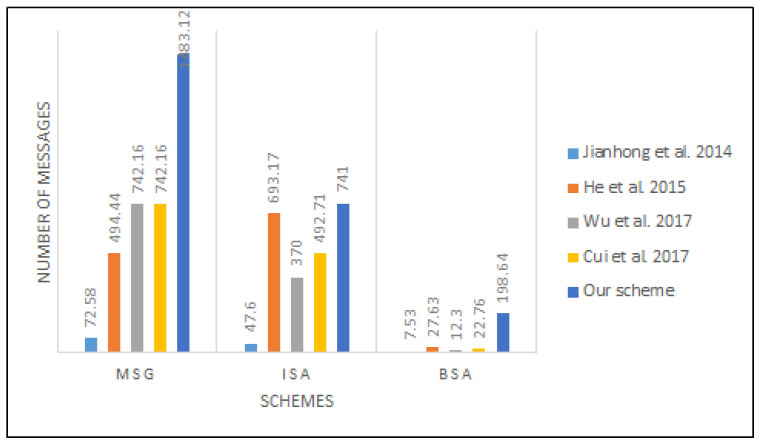
The computation cost’s speed.

To satisfy the privacy requirements in terms of identity preserving and unlinkability, the scheme uses the elliptic curve operations. For example, the proposed scheme randomly selects unused pseudonym-IDs for signing a message from a pseudonym-ID list to avoid the adversary linking two or more messages sent from the same source, while the proposed scheme computes a new pseudonym-ID to sign each message. Thereby the computing cost will increase. For He et al.’s scheme [25], we can conclude (1 − 0.0020184)/0.0020184 ≈ 494.44, (1 − 0.0020236)/0.0020236 ≈ 693.17 and (1 − (0.6718 × 50 + 1.3405)/1000)/0.0349305 ≈ 27.63 that signed messages, individual signature authentication, and batch signature authentication in 1 s, respectively. For the proposed scheme, we can conclude (1 − 0.0006738)/0.0006738 ≈ 1483.12, (1 − 0.0013477)/0.0013477 ≈ 741 and (1 − (0.0737 × 50 + 1.3467)/1000)/0.0050317) ≈ 198.46 that signed messages, individual signature authentication, and batch signature authentication in 1 s, respectively. A similar method was used for the schemes for comparative purposes, and the result is shown in Figure 10.

As presented in Table 4, the computation cost of the proposed scheme decreased by (2.0184 − 0.6738)/2.0184 ≈ 66.7%, (2.0236 − 1.3477)/2.0236 ≈ 33.4% and ((0.6718 × 50 +1.3405) − (0.0737 × 50 + 1.3467))/(0.6718 × 50 + 1.3405) ≈ 90.3% for MSG, ISA, and BSA, respectively, compared to the He et al. scheme [25]. Table 5 presents the performance of the proposed scheme against the existing schemes for MSG, ISA, and BSA. The computational result shows that the elliptic curve used in the proposed scheme could handle the very fast pseudonym-changing process in signing and verifying messages in VANETs.

**Figure 11:**
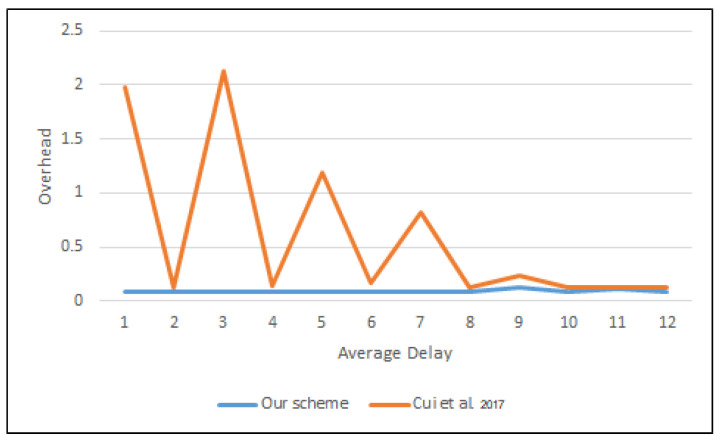
Average delay to a sign message in OMNeT++.

**Figure 12:**
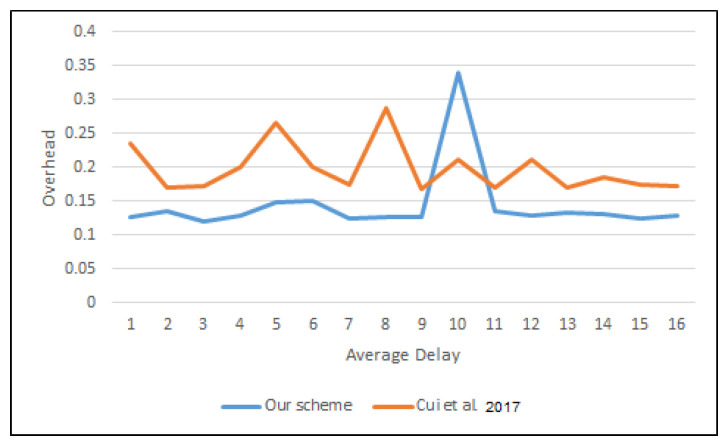
Average delay to verify a message in OMNeT++.

Hence, the total time was based on the execution time of each operation. The Elapsed Time (*ET*) between the exit and entrance to the SPS layer is the overhead cost.

(8) $$ET=\frac{1}{M}\sum_{i=1}^{n}M\left(T_{out}^{i}-T_{in}^{i}\right)$$

where *M* is the number of messages and $T_{out}^{i}$ and $T_{in}^{i}$ are the exit and entrance times of message *i*, respectively. Figure 11 and Figure 12 depict the average time to sign and verify the message between the proposed scheme and that of Cui et al. [30].

### 7.3. Communication Cost Analysis and Comparison

This subsection evaluates and compares the communication costs between the proposed scheme and the existing schemes. Based on the experiment by He et al. [25], let the sizes of the elements in $G_{1}$ and *G* be 128 bytes and 40 bytes, respectively. Besides, let the elements in $Z_{q}^{*}$, the size of the timestamp, and the output of a hash function be 20 bytes, 4 bytes, and 20 bytes, respectively.

In the scheme of He et al. [25], the format of the message signature tuple <$pid_{in}$, $m_{i}$, $T_{i}$, $R_{i}$, $\sigma_{i}$>, due to the $pid_{il}^{1}$, $pid_{il}^{2}$ and $\sigma_{i}\in Z_{q}^{*}$, $R_{i}\in G$ and one timestamp; thus, the full size is 40 × 3 + 20 + 4 = 144 bytes. In the proposed scheme, the vehicle sends the message signature tuple {$pid_{in}$, $m_{i}$, *T*, $\sigma_{i}$} with size 40 +20 × 3 + 8 = 104 bytes. The metrics for the other schemes were also measured using the same procedure. Table 6 lists the communication cost comparison of our scheme with the other schemes.

## 8. Conclusions

This paper proposed a secure pseudonym-based conditional privacy-preservation authentication scheme to secure V2V and V2I communications in VANETs. The proposed scheme eliminates the dependency on RSU-only authentication by using many pseudo-IDs with corresponding signature keys from the TA, therefore allowing each vehicle to authenticate the received messages directly. The proposed scheme is resistant to insider attacks as the TA can revoke rogue vehicles’ certificates, preventing them from continuously broadcasting fake messages. The security analysis proved that the proposed scheme under the random oracle model is secure, and it also satisfies the security and privacy requirements. Since the proposed approach uses ECC, its computation cost overhead is lower than other related bilinear pair-based approaches. Future work could include the analysis and performance measurement of the proposed approach in terms of latency, average delay, and throughput using network simulators, such as OMNeT++, and road traffic simulators, such as SUMO. Besides, the future work will also include the design of an authentication scheme based on fog computing that does not use ECC in 5G-enabled vehicular networks.

## Figures and Tables

**Table 1:** Notations and their description.

Notations	Descriptions
a,b	Two large prime numbers
*p*	A large prime number
*E*	The elliptic curve
*G*	The additive group based on E
*P*	The base generator P∈ G
$h_{1},h_{2},h_{3}$	The three functions of the one-way hash
$ID_{VI}$, PW	The identity and password of the vehicle
*s*, $P_{pub}$	The private and public key of the system
$pid_{il}^{1}$, $pid_{il}^{2}$	The pseudo-identity of the vehicle
⊕	The XOR operator
$LPID_{i}$	The list of pseudo-identities
$\zeta_{l}$	The random secret value
‖	The concatenation operation
$LSK_{i}$	The list of signature keys

**Table 2:** Comparison of the security issues.

	[17,18,19,20,21,22,23,24]	[25,26,27,28,29]	[30,31]	Proposed
SC-1	✓	✗	✗	✗
SC-2	✓	✓	✗	✗
SC-3	✗	✗	✓	✗

**Table 3:** Simulation experiment parameters.

Parameters	Value
Simulation time	200 s
Playground size	x = 3463 m, y = 4270 m and z = 50 m
Mac layer	IEEE 1609.4
Physical layer	IEEE 802.11p
Maximum transmission	20 mW
Bit rate	6 Mbps
Number of vehicles	500
Minimum speed	30 Km/H
Maximum speed	60 Km/H

**Table 4:** The computation cost of the five authentication schemes.

Scheme	MSG	ISA	BSA
Jianhong et al. [24]	$6T_{b-m}+2T_{b-a}+1T_{mtp}+4T_{h}\approx 13.59$	$3T_{bp}+2T_{b-m}+1T_{b-a}+3T_{h}\approx 20.5774$	$3T_{bp}+\left(n+1\right)T_{b-m}+\left(2n\right)T_{b-sm}+\left(3n-2\right)T_{b-a}+\left(3n\right)T_{h}\approx 1.966n+18.9772$
He et al. [25]	$3T_{e-m}+3T_{h}\approx 2.0184$	$3T_{e-m}+2T_{e-a}+2T_{h}\approx 2.0236$	$\left(n+2\right)T_{e-m}+\left(2n\right)T_{e-sm}+\left(2n-1\right)T_{e-a}+\left(2n\right)T_{h}\approx 0.6718n+1.3405$
Wu et al. [28]	$2T_{e-m}+2T_{h}\approx 1.3456$	$4T_{e-m}+2T_{h}+2T_{e-a}\approx 2.6954$	$\left(2n+2\right)T_{e-m}+\left(2n\right)T_{e-sm}+\left(2n+2\right)T_{e-a}+\left(2n\right)T_{h}\approx 1.4786n+1.3498$
Cui et al. [30]	$2T_{e-m}+2T_{h}\approx 1.3456$	$3T_{e-m}+7T_{h}+1T_{e-a}\approx 2.0255$	$\left(n+2\right)T_{e-m}+\left(2n\right)T_{e-sm}+\left(n+1\right)T_{e-a}+\left(7n\right)T_{h}\approx 0.8149n+1.3467$
Our scheme	$1T_{e-m}+2T_{h}\approx 0.6738$	$2T_{e-m}+1T_{h}+1T_{e-a}\approx 1.3477$	$2T_{e-m}+\left(2n\right)T_{e-sm}+\left(n+1\right)T_{e-a}+\left(n\right)T_{h}\approx 0.0737n+1.3467$

**Table 5:** Improvement of computation overhead comparison.

Scheme	MSG	ISA	BSA (50 Messages)
Jianhong et al. [24]	96.9%	94.5%	97.8%
He et al. [25]	66.7%	33.4%	90.3%
Wu et al. [28]	49.9%	50%	94.9%
Cui et al. [30]	49%	33.3%	90.2%

**Table 6:** Comparison of communication costs.

Schemes	Single Message (Bytes)	Batch Messages (Bytes)
Jianhong et al. [24]	388	388 n
He et al. [25]	144	144 n
Wu et al. [28]	148	148 n
Cui et al. [30]	84	84 n
Our scheme	104	104 n

## Data Availability

Data sharing not applicable.
